# Machine learning-based scoring model for predicting mortality in ICU-admitted ischemic stroke patients with moderate to severe consciousness disorders

**DOI:** 10.3389/fneur.2025.1534961

**Published:** 2025-03-18

**Authors:** Zhou Zhou, Bo Chen, Zhao-Jun Mei, Wei Chen, Wei Cao, En-Xi Xu, Jun Wang, Lei Ye, Hong-Wei Cheng

**Affiliations:** ^1^Department of Neurosurgery, The First Affiliated Hospital of Anhui Medical University, Hefei, China; ^2^Department of Neurosurgery, Affiliated People’s Hospital of Jiangsu University, Jiangsu, China; ^3^Department of Neurology, Affiliated People’s Hospital of Jiangsu University, Jiangsu, China

**Keywords:** ischemic stroke, consciousness disorders, scoring model, machine learning, mortality

## Abstract

**Background:**

Stroke is a leading cause of mortality and disability globally. Among ischemic stroke patients, those with moderate to severe consciousness disorders constitute a particularly high-risk subgroup. Accurate predictive models are essential for guiding clinical decisions in this population. This study aimed to develop and validate an automated scoring system using machine learning algorithms for predicting short-term (3- and 7-day) and relatively long-term (30- and 90-day) mortality in this population.

**Methods:**

This retrospective observational study utilized data from the MIMIC-IV database, including 648 ischemic stroke patients with Glasgow Coma Scale (GCS) scores ≤12, admitted to the ICU between 2008 and 2019. Patients with GCS scores indicating speech dysfunction but clear consciousness were excluded. A total of 47 candidate variables were evaluated, and the top six predictors for each mortality model were identified using the AutoScore framework. Model performance was assessed using the area under the curve (AUC) from receiver operating characteristic (ROC) analyses.

**Results:**

The median age of the cohort was 76.8 years (IQR, 64.97–86.34), with mortality rates of 8.02% at 3 days, 18.67% at 7 days, 33.49% at 30 days, and 38.89% at 90 days. The AUCs for the test cohort’s 3-, 7-, 30-, and 90-day mortality prediction models were 0.698, 0.678, 0.724, and 0.730, respectively.

**Conclusion:**

We developed and validated a novel machine learning-based scoring tool that effectively predicts both short-term and relatively long-term mortality in ischemic stroke patients with moderate to severe consciousness disorders. This tool has the potential to enhance clinical decision-making and resource allocation for these patients in the ICU.

## Introduction

Stroke, including both ischemic and hemorrhagic types, remains one of the leading causes of mortality and long-term disability worldwide (1). Stroke mortality is projected to increase by 50% from 2020 to 2050 (2), significantly adding to the disease burden. The burden is particularly severe among patients who experience both severe ischemic stroke and consciousness disorders (3, 4), involving prolonged hospital stays, intensive rehabilitation efforts, and significant caregiver support (5). Consciousness disorders encompass a range of conditions, including coma, vegetative state, and minimally conscious state (6, 7), and are associated with significantly worse prognoses compared to ischemic stroke patients without consciousness disorders (8).

In this study, we focus on a distinct and challenging subgroup: ischemic stroke patients with moderate to severe consciousness disorders (GCS ≤ 12) at admission, excluding those with a GCS score of 4-1-6 or 4-2-6, as they are classified as having speech dysfunction with clear consciousness (9, 10). All these severe ischemic stroke patients were admitted to the ICU (11).

Patients in this category are typically incapable of independently deciding on interventions such as mechanical ventilation, artificial nutrition, surgical decompression, or even the withdrawal of life-sustaining treatment. In many severe stroke cases, however, physicians and patient surrogates must make decisions under conditions of prognostic uncertainty and ambiguous definitions of acceptable outcomes (12). Accurate prediction of outcomes in these patients is essential for guiding clinical decisions, managing resources, and providing appropriate counseling for patients’ families. Prognostic models that accurately predict outcomes for patients with severe stroke are currently insufficient. Traditional assessment tools, such as the GCS and the Modified Rankin Scale (mRS), often overlook the complexities inherent in these patients’ conditions. Moreover, these models tend to rely on static clinical evaluations and do not take advantage of the massive data available from modern healthcare databases. Recent advancements in machine learning (ML) have shown potential in developing more precise and individualized prognostic models (13, 14). ML techniques can analyze large datasets to identify patterns often missed by traditional methods, enhancing prognostic accuracy for patients (15, 16). Despite its potential, research applying machine learning to predict outcomes in severe ischemic stroke patients remains limited. This gap underscores the need for innovative approaches to improve prognostic accuracy in this high-risk population.

Therefore, the primary objective of this study is to develop an automated scoring model using machine learning techniques to estimate mortality for severe ischemic stroke patients with moderate to severe consciousness disorders. By enhancing the interpretability and accuracy of the predictive model, we aim to facilitate its integration into clinical workflows and decision-making processes.

## Methods

### Study population

This study is a retrospective observational analysis, and data were extracted from the Medical Information Mart for Intensive Care IV (MIMIC-IV) database. The MIMIC-IV database includes records of more than 40,000 patients admitted to the intensive care units at Beth Israel Deaconess Medical Center between 2008 and 2019 (17). This database contains detailed patient information, including demographic characteristics, vital signs, laboratory test results, prescribed medications, and other relevant data. Author Zhou Zhou secured permission to access the dataset (Record ID 11493928) and was responsible for data extraction. Institutional review boards at the Massachusetts Institute of Technology (MIT) and Beth Israel Deaconess Medical Center (BIDMC) approved the project and issued a waiver for informed consent.

**Figure 1:**
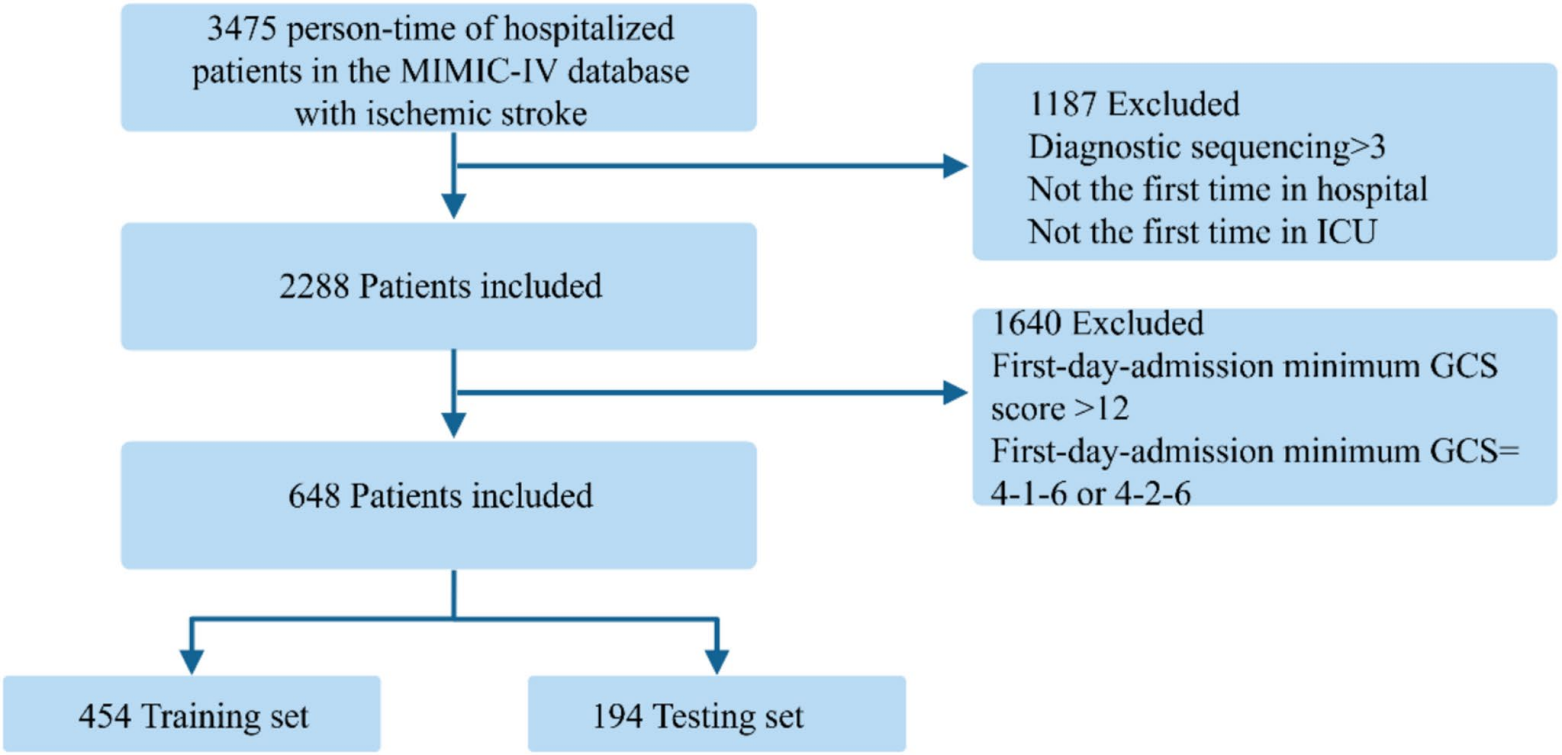
Flow diagram of patients included in the analysis.

A total of 3,475 person-time records of hospitalized patients with ischemic stroke were included from the MIMIC-IV database. Ischemic stroke was diagnosed at ICU admission based on the International Classification of Diseases, Ninth Revision (ICD-9) and Tenth Revision (ICD-10) codes. ICD-9: 43,301, 43,311, 43,321, 43,331, 43,381, 43,391, 43,401, 43,411, and 43,491; ICD-10: I63. Patients with a diagnostic sequence greater than 3 or those who were not making their first hospital or ICU admission were excluded. Additionally, we analyzed only patients whose minimum GCS score on the first day of admission was ≤12, excluding those with GCS scores of 4-1-6 or 4-2-6 because these patients typically exhibit verbal dysfunction without impaired consciousness, which could introduce heterogeneity into the cohort. A total of 648 patients met the inclusion criteria and were randomly divided into a training set (70%) and a testing set (30%). The patient screening flow diagram is displayed in Figure 1.

### Variables extraction

The first-time data on baseline characteristics within the first 24 h of ICU admission were extracted from the MIMIC-IV database, including demographics, vital signs, laboratory tests, comorbidities, and scoring systems.

### Outcome

Short-term mortality was defined as death within 3 and 7 days after admission, whereas relatively long-term mortality was defined as death occurring within 30 and 90 days after admission. Patient mortality information for discharged patients was obtained from the US Social Security Death Index and recorded in the MIMIC-IV database.

### Statistical analysis

Data were presented as the mean with standard deviation (SD) or median with interquartile range (IQR) for continuous variables and as quantity and frequency (%) for categorical variables. Continuous variables were compared using Student’s t-test (normal distribution) or Mann–Whitney U-Test (normal distribution with heteroscedasticity or non-normal distribution) and categorical variables were compared using the Pearson chi-square test (expected counts T ≥ 5 and total sample size *n* ≥ 40) or Fisher’s exact test (expected counts T < 5 and total sample size *n* ≥ 40). Multiple interpolation was used to fill in missing values (less than 20%). The confidence level was set at *α* = 0.05. All statistical analyses were conducted using R 4.3.1. *p* < 0.05 was considered statistically significant.

**Figure 2:**
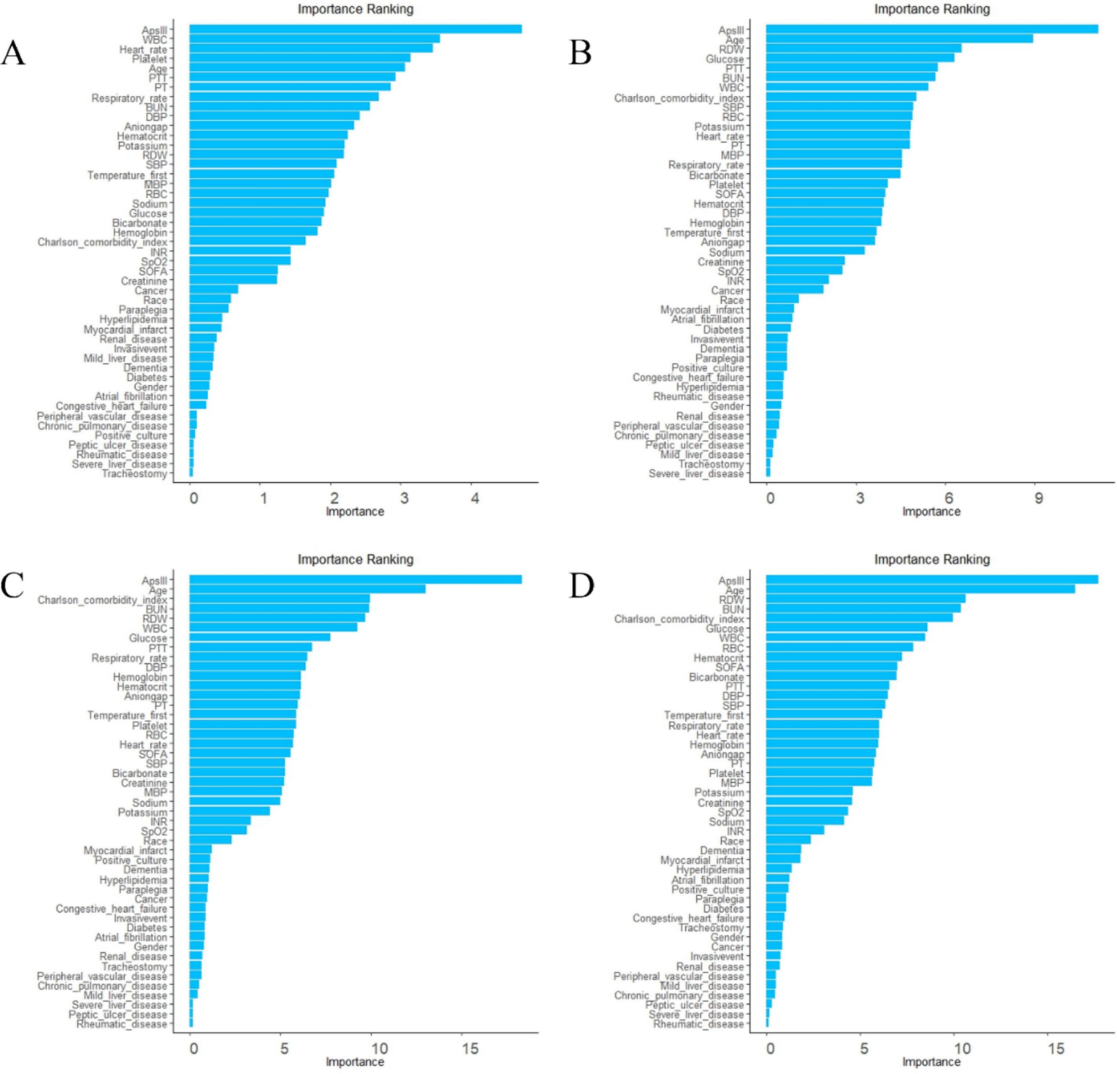
Ranking list of variable importance based on random forest. **(A)** variable ranking list for 3-day death. **(B)** Variable ranking list for 7-day death. **(C)** Variable ranking list for 30-day death. **(D)** Variable ranking list for 90-day death.

**Figure 3:**
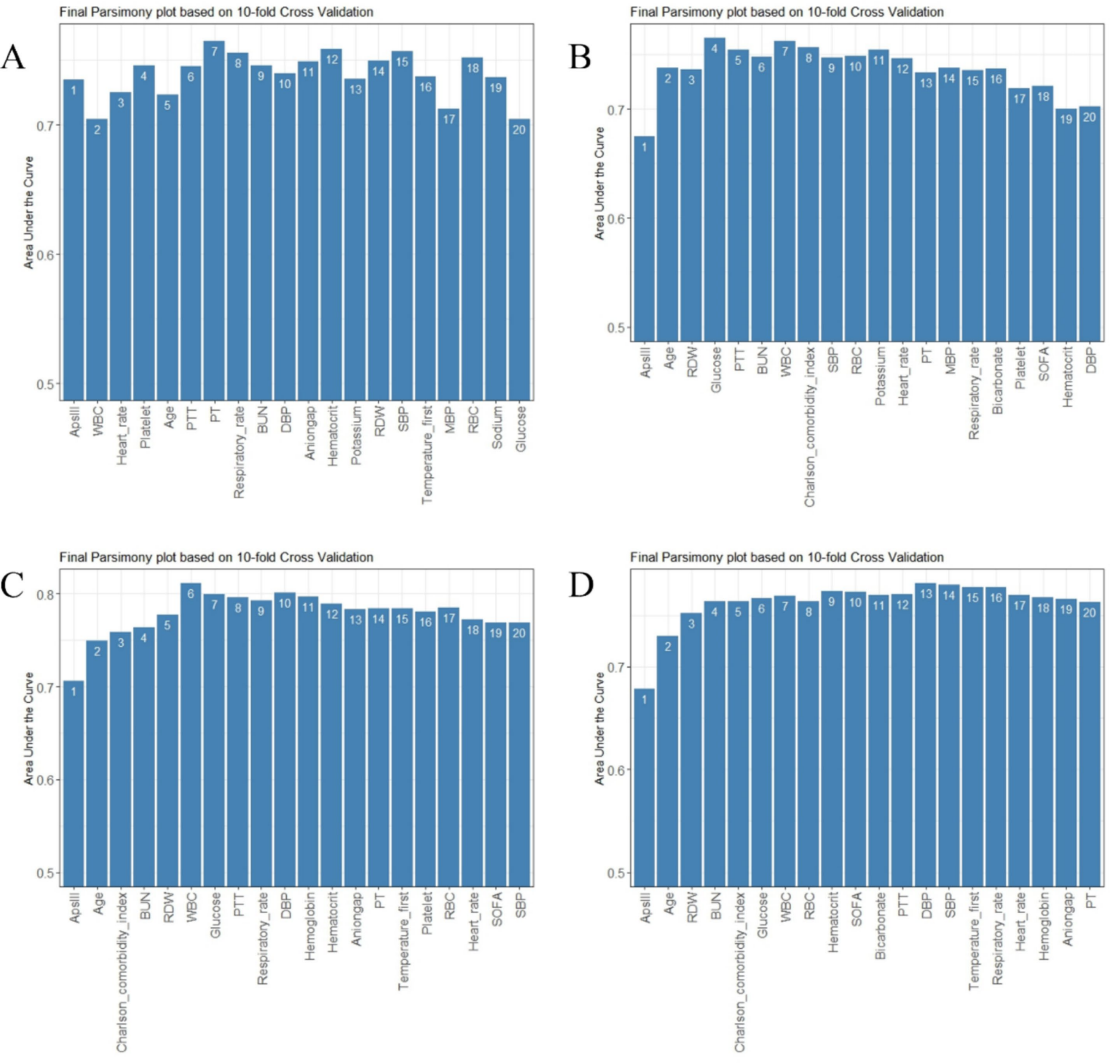
Parsimony plot of variables. **(A)** Parsimony plot for 3-day death. **(B)** Parsimony plot for 7-day death. **(C)** Parsimony plot for 30-day death. **(D)** Parsimony plot for 90-day death.

**Figure 4:**
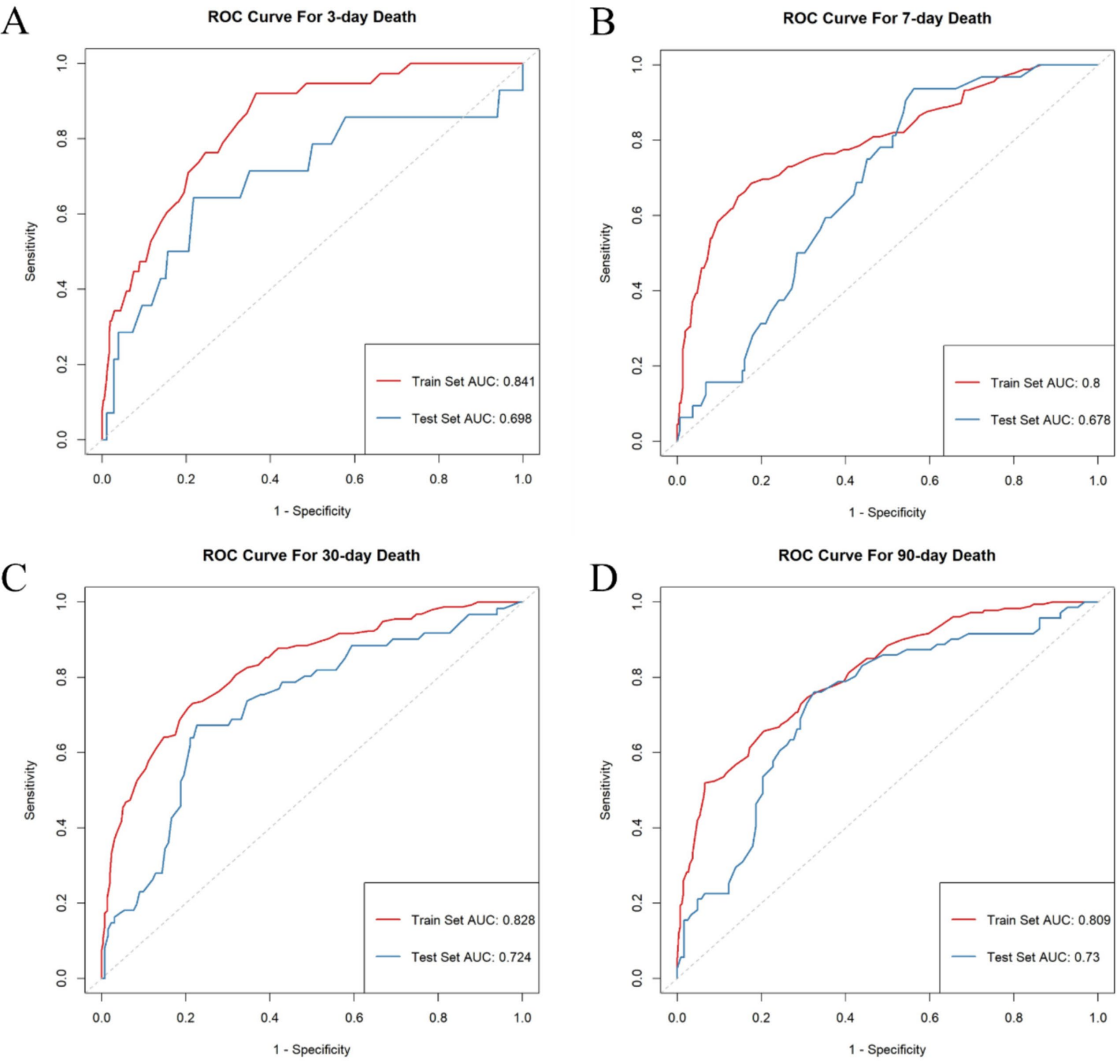
Receiver operating characteristic curves for each model. **(A)** ROC curve for 3-day death. **(B)** ROC curve for 7-day death. **(C)** ROC curve for 30-day death. **(D)** ROC curve for 90-day death.

The AutoScore framework is a systematic and automated clinical score generator, it can generate parsimonious sparse-score risk models from electronic health records (EHRs) or other types of medical data based on machine learning and regression modeling (18). In this study, the AutoScore framework was implemented to construct validated risk-scoring models capable of predicting mortality at intervals of 3, 7, 30, and 90 days. Firstly, the AutoScore binary program was used to identify the top-ranking predictors based on machine learning algorithms, and a parsimonious list of variables for the final scoring model was selected using a parsimony plot. Secondly, six key variables were selected as the most influential variables, and initial scores were generated based on these variables (Figures 2, 3). Thirdly, the data-driven cutoff values for each continuous variable were refined to generate the final scoring system, which was then evaluated using the testing dataset. The final prognostic model scoring chart is presented in Table 1. The predictive performance of the scoring models was quantified using the AUC within the ROC analysis (Figure 4). Sensitivity, specificity, positive predictive value, and negative predictive value were calculated at the optimal threshold and reported with a 95% confidence interval (Tables 2, 3).

**Table 1:** Scoring models according to outcomes.

Variable	3d-death score		7d-death score		30d-death score		90d-death score	
	Interval	Point	Interval	Point	Interval	Point	Interval	Point
ApsIII								
	<40	6	<40	0	<50	0	<40	1
	[40,50)	7	[40,50)	5	[50,64)	16	[40,50)	0
	[50,64)	0	[50,64)	14	≥ 64	24	[50,64)	15
	≥ 64	17	≥ 64	24			≥ 64	22
Age, years								
	<65	0	<65	0	<65	0	<65	0
	[65,86)	3	[65,76)	8	[65,76)	14	[65,86)	13
	≥ 86	10	[76,86)	5	[76,86)	13	≥ 86	28
			≥ 86	24	≥ 86	26		
RDW, %								
			<13.2	0	<13.2	0	<13.2	3
			[13.2,15.1)	8	[13.2,13.9)	10	[13.2,13.9)	13
			≥ 15.1	16	[13.9,15.1	0	[13.9,15.1	0
					≥ 15.1	13	≥ 15.1	16
BUN, mg/dL								
			<14	0	<14	0	<14	0
			[14,18)	5	[14,18)	5	[14,18)	6
			[18,24)	3	[18,24)	0	[18,24)	3
			≥ 24	5	≥ 24	10	≥ 24	12
WBC, K/μL								
	<7.9	0			<10.2	0		
	[7.9,10.2)	7			[10.2,13.5)	13		
	[10.2,13.5)	20			≥ 13.5	15		
	≥ 13.5	24						
PTT, sec								
	<25.6	0	<25.6	3				
	[25.6,32.6)	7	[25.6,28.2)	11				
	≥ 32.6	10	[28.2,32.6)	0				
			≥ 32.6	5				
Glucose, mg/dL								
			<107	0			<107	3
			[107,160)	8			[107,126)	0
			≥ 160	19			[126,160)	4
							≥ 160	13
Charlson_comorbidity_index								
					<5	12	<5	7
					[5,7)	0	[5,7)	0
					[7,8)	9	[7,8)	3
					≥ 8	10	≥ 8	9
Heart_rate, bmp								
	<71	0						
	[71,82)	1						
	[82,96)	4						
	≥ 96	16						
Platelet, K/μL								
	<158	23						
	[158,210)	19						
	[210,276)	21						
	≥276	0						

**Table 2:** Conversion tables with predictive performance measures for specific score thresholds based on the scoring models of 3d-death, 7d-death, 30d-death, and 90d-death.

Score cut-off [≥]	Predicted Risk [≥]	Percentage of patients (%)	Accuracy (95% CI)	Sensitivity (95% CI)	Specificity (95% CI)	PPV (95% CI)	NPV (95% CI)
3d-death scoring model							
20	2%	96	9.8% (7.2–12.4%)	92.9% (78.6–100%)	3.3% (1.1–6.1%)	7% (5.9–7.6%)	85.7% (50–100%)
40	4.1%	76	29.4% (23.7–35.6%)	85.7% (64.3–100%)	25% (18.9–31.7%)	8.3% (6.2–9.8%)	95.9% (89.6–100%)
60	8.1%	35	67% (60.3–73.7%)	64.3% (35.7–85.7%)	67.2% (60.6–73.9%)	13.3% (8.2–18.3%)	96.1% (93.3–98.4%)
80	15.4%	6	91.2% (88.1–94.3%)	28.6% (7.1–57.1%)	96.1% (93.3–98.9%)	36.4% (11.1–66.7%)	94.5% (93–96.6%)
7d-death scoring model							
20	8.1%	88	28.4% (24.2–33%)	100% (100–100%)	14.2% (9.3–19.8%)	18.7% (17.9–19.8%)	100% (100–100%)
40	13.5%	56	53.6% (47.4–60.3%)	78.1% (62.5–90.6%)	48.8% (42–56.2%)	23.2% (19.1–27.4%)	92% (86.9–96.7%)
60	22.3%	23	71.1% (65.5–77.3%)	31.2% (15.6–46.9%)	79% (72.8–85.2%)	22.9% (13–34.1%)	85.4% (82.5–88.5%)
80	35.8%	5	82% (78.9–84.5%)	9.4% (0–18.8%)	96.3% (93.2–98.8%)	33.3% (0–66.7%)	84.2% (83–85.9%)
30d-death scoring model							
20	11.3%	91	38.7% (34.5–42.8%)	96.7% (91.8–100%)	12% (6.8–18%)	33.5% (31.8–35.3%)	89.5% (72.2–100%)
40	22.8%	61	58.8% (52.1–65.5%)	82% (72.1–90.2%)	48.1% (39.1–56.4%)	42.1% (37.1–47.3%)	85.4% (78.2–92.2%)
60	40.7%	30	72.2% (66–77.8%)	54.1% (41–67.2%)	80.5% (73.7–87.2%)	56.1% (45.8–66.7%)	79.4% (74.6–84.3%)
80	64.4%	6	71.6% (68.6–74.7%)	14.8% (6.6–24.6%)	97.7% (94.7–100%)	75% (50–100%)	71.4% (69.4–73.9%)
90d-death scoring model							
20	15.7%	89	42.8% (38.7–47.4%)	93% (87.3–98.6%)	13.8% (8.1–20.3%)	38.4% (36.2–40.7%)	77.8% (60–94.1%)
40	29.3%	58	66% (59.8–72.2%)	83.1% (73.2–91.5%)	56.1% (48–65%)	52.2% (46.8–58%)	85.2% (78–91.5%)
60	48%	29	68.6% (62.9–74.7%)	46.5% (35.2–57.7%)	81.3% (74.8–87.8%)	59.3% (49–70.2%)	72.5% (68.1–77.4%)
80	68.1%	7	67.5% (63.9–71.1%)	15.5% (7–23.9%)	97.6% (94.3–100%)	80% (55.6–100%)	66.7% (64.5–69.1%)

PPV positive predictive value, NPV negative predictive value. Varying cutoffs of predicted risk based on the scoring models of 3d-death, 7d-death, 30d-death, and 90d-death, the proportion of patients stratified for the outcomes, and the corresponding sensitivity, specificity, positive and negative predictive values on the testing cohort.

**Table 3:** Predictive model performance.

	Training set	Testing set
3d-death scoring model		
AUC	0.8406 (0.7806–0.9006)	0.698 (0.5219–0.8741)
Best score threshold	≥55	≥66
Sensitivity	0.9211	0.6429 (0.4286–0.8571)
Specificity	0.6346	0.7833 (0.7222–0.8444)
PPV	0.1872	0.1905 (0.12–0.2708)
NPV	0.9888	0.9662 (0.9429–0.9866)
7d-death scoring model		
AUC	0.8004 (0.7449–0.856)	0.678 (0.5919–0.764)
Best score threshold	≥57	≥35
Sensitivity	0.6854	0.9375 (0.8438–1)
Specificity	0.8247	0.4383 (0.3642–0.5123)
PPV	0.488	0.2479 (0.2177–0.2793)
NPV	0.9149	0.9733 (0.9318–1)
30d-death scoring model		
AUC	0.8281 (0.7879–0.8684)	0.7245 (0.6462–0.8027)
Best score threshold	≥53	≥56
Sensitivity	0.7308	0.6721 (0.541–0.7869)
Specificity	0.7852	0.7744 (0.7068–0.8496)
PPV	0.6404	0.5789 (0.4998–0.6769)
NPV	0.8478	0.8385 (0.7879–0.888)
90d-death scoring model		
AUC	0.8095 (0.7696–0.8493)	0.7297 (0.6555–0.8039)
Best score threshold	≥61	≥46
Sensitivity	0.5193	0.7606 (0.662–0.8592)
Specificity	0.9341	0.6748 (0.5935–0.7561)
PPV	0.8393	0.573 (0.5056–0.6471)
NPV	0.7456	0.8302 (0.77–0.8901)

Performance indicators (Best score threshold, Sensitivity, Specificity, PPV, NPV) based on best score threshold, values in parentheses are 95% confidence intervals.

## Results

### Baseline characteristics

Patient characteristics for the entire population, as well as the training and testing cohorts, are detailed in Table 4. In the total cohort, the median length of stay in the ICU was 3.055 days [IQR 1.807, 6.602], and the median hospital stay was 8.990 days [IQR 5.108, 15.532]. Of the patients, 493 (76.08%) were discharged alive, while 155 (23.92%) did not survive.

**Table 4:** The baseline level of 648 patients.

Categories	Level	Overall	Training set	Testing set	*p*	Test
Number		648	454	194		
LOS_ICU, days (median [IQR])		3.055 [1.807, 6.602]	3.170 [1.778, 6.835]	2.880 [1.815, 5.822]	0.4137	Non-norm
LOS_Hospital, days (median [IQR])		8.990 [5.108, 15.532]	9.001 [5.071, 15.586]	8.960 [5.353, 14.936]	0.9793	Non-norm
Hospital_expire_flag (%)	Live	493 (76.08)	342 (75.33)	151 (77.84)	0.5592	
	Dead	155 (23.92)	112 (24.67)	43 (22.16)		
Status_3d (%)	Live	596 (91.98)	416 (91.63)	180 (92.78)	0.736	
	Dead	52 (8.02)	38 (8.37)	14 (7.22)		
Status_7d (%)	Live	527 (81.33)	365 (80.40)	162 (83.51)	0.4122	
	Dead	121 (18.67)	89 (19.60)	32 (16.49)		
Status_30d (%)	Live	431 (66.51)	298 (65.64)	133 (68.56)	0.5287	
	Dead	217 (33.49)	156 (34.36)	61 (31.44)		
Status_90d (%)	Live	396 (61.11)	273 (60.13)	123 (63.40)	0.4877	
	Dead	252 (38.89)	181 (39.87)	71 (36.60)		
Gender (%)	Female	386 (59.57)	263 (57.93)	123 (63.40)	0.2253	
	Male	262 (40.43)	191 (42.07)	71 (36.60)		
Age, years (median [IQR])		76.808 [64.969, 86.338]	75.897 [65.250, 86.058]	77.785 [64.619, 86.605]	0.8302	Non-norm
Race (%)	Asian	24 (3.70)	13 (2.86)	11 (5.67)	0.2964	
	Black	65 (10.03)	44 (9.69)	21 (10.82)		
	White	372 (57.41)	269 (59.25)	103 (53.09)		
	Hispanic/latino	23 (3.55)	14 (3.08)	9 (4.64)		
	Other	164 (25.31)	114 (25.11)	50 (25.77)		
Aniongap, mEq/L (median [IQR])		15.000 [13.000, 17.000]	15.000 [13.000, 17.000]	15.000 [13.000, 16.000]	0.1202	Non-norm
Bicarbonate, mEq/L (median [IQR])		23.000 [21.000, 26.000]	23.000 [21.000, 26.000]	23.000 [21.000, 25.000]	0.7844	Nonn-orm
BUN, mg/dL (median [IQR])		18.000 [14.000, 25.000]	18.000 [14.000, 24.000]	18.000 [13.000, 26.750]	0.7547	Non-norm
Creatinine, mg/dL (median [IQR])		0.900 [0.700, 1.100]	0.900 [0.700, 1.100]	0.900 [0.700, 1.200]	0.8633	Non-norm
Glucose, mg/dL (median [IQR])		127.000 [108.000, 160.000]	126.000 [107.000, 159.750]	131.500 [109.000, 162.250]	0.4904	Non-norm
Sodium, mEq/L (median [IQR])		140.000 [137.000, 142.000]	140.000 [137.000, 142.000]	140.000 [137.000, 142.000]	0.847	Non-norm
Potassium, mEq/L (median [IQR])		4.000 [3.700, 4.400]	4.000 [3.700, 4.400]	4.100 [3.725, 4.500]	0.0354	Non-norm
Hematocrit, % (median [IQR])		35.700 [32.200, 39.800]	35.550 [32.125, 39.800]	36.050 [32.600, 40.025]	0.5376	Non-norm
Hemoglobin, g/dL (mean (SD))		11.838 (2.082)	11.806 (2.027)	11.912 (2.207)	0.5524	
Platelet, K/μL (median [IQR])		210.000 [161.000, 277.000]	210.000 [158.000, 276.000]	209.000 [167.000, 278.500]	0.4408	Non-norm
WBC, K/μL (median [IQR])		10.300 [8.000, 13.500]	10.200 [7.900, 13.500]	10.350 [8.500, 13.300]	0.3311	Non-norm
RBC, m/μL (median [IQR])		4.010 [3.507, 4.440]	3.995 [3.500, 4.408]	4.100 [3.572, 4.488]	0.2766	Non-norm
RDW, % (median [IQR])		14.000 [13.200, 15.100]	13.900 [13.200, 15.100]	14.050 [13.300, 14.900]	0.8691	Non-norm
INR (median [IQR])		1.200 [1.100, 1.300]	1.200 [1.100, 1.300]	1.200 [1.100, 1.300]	0.4089	Non-norm
PT, sec (median [IQR])		12.900 [11.800, 14.400]	12.800 [11.700, 14.300]	13.000 [12.000, 14.700]	0.1765	Non-norm
PTT, sec (median [IQR])		28.300 [25.600, 33.025]	28.200 [25.600, 32.575]	29.000 [25.525, 34.675]	0.1547	Non-norm
Heart rate, bmp (median [IQR])		82.000 [72.000, 96.000]	82.000 [71.000, 96.000]	83.000 [72.250, 93.750]	0.7242	Non-norm
SBP, mmHg (median [IQR])		141.000 [123.000, 157.000]	140.000 [121.000, 156.750]	145.500 [127.000, 158.750]	0.0519	Non-norm
DBP, mmHg (median [IQR])		74.000 [63.000, 88.000]	73.000 [63.000, 86.750]	76.500 [65.000, 91.000]	0.0357	Non-norm
MBP, mmHg (median [IQR])		92.000 [81.000, 106.000]	91.000 [81.000, 104.000]	96.500 [83.000, 108.750]	0.0133	Non-norm
Respiratory rate, insp/min (median [IQR])		18.000 [16.000, 22.000]	18.000 [16.000, 22.000]	19.000 [16.000, 22.750]	0.489	Non-norm
Temperature, °C (median [IQR])		36.720 [36.440, 37.073]	36.720 [36.440, 37.110]	36.720 [36.440, 37.060]	0.6708	Non-norm
SPO2, % (median [IQR])		98.000 [96.000, 100.000]	98.000 [96.000, 100.000]	98.000 [96.000, 100.000]	0.2986	Non-norm
Atrial fibrillation (%)	No	365 (56.33)	260 (57.27)	105 (54.12)	0.5139	
	Yes	283 (43.67)	194 (42.73)	89 (45.88)		
Hyperlipidemia (%)	No	342 (52.78)	237 (52.20)	105 (54.12)	0.7168	
	Yes	306 (47.22)	217 (47.80)	89 (45.88)		
Myocardial infarct (%)	No	561 (86.57)	393 (86.56)	168 (86.60)	1	
	Yes	87 (13.43)	61 (13.44)	26 (13.40)		
Congestive heart failure (%)	No	499 (77.01)	352 (77.53)	147 (75.77)	0.6997	
	Yes	149 (22.99)	102 (22.47)	47 (24.23)		
Peripheral vascular disease (%)	No	574 (88.58)	402 (88.55)	172 (88.66)	1	
	Yes	74 (11.42)	52 (11.45)	22 (11.34)		
Dementia (%)	No	601 (92.75)	421 (92.73)	180 (92.78)	1	
	Yes	47 (7.25)	33 (7.27)	14 (7.22)		
Chronic pulmonary disease (%)	No	538 (83.02)	371 (81.72)	167 (86.08)	0.2146	
	Yes	110 (16.98)	83 (18.28)	27 (13.92)		
Rheumatic disease (%)	No	628 (96.91)	438 (96.48)	190 (97.94)	0.4606	
	Yes	20 (3.09)	16 (3.52)	4 (2.06)		
Peptic ulcer disease (%)	No	639 (98.61)	446 (98.24)	193 (99.48)	0.2917	Exact
	Yes	9 (1.39)	8 (1.76)	1 (0.52)		
Mild liver disease (%)	No	625 (96.45)	434 (95.59)	191 (98.45)	0.1165	
	Yes	23 (3.55)	20 (4.41)	3 (1.55)		
Paraplegia (%)	No	270 (41.67)	189 (41.63)	81 (41.75)	1	
	Yes	378 (58.33)	265 (58.37)	113 (58.25)		
Renal disease (%)	No	549 (84.72)	384 (84.58)	165 (85.05)	0.9736	
	Yes	99 (15.28)	70 (15.42)	29 (14.95)		
Severe liver disease (%)	No	640 (98.77)	447 (98.46)	193 (99.48)	0.4465	Exact
	Yes	8 (1.23)	7 (1.54)	1 (0.52)		
Diabetes (%)	No	450 (69.44)	320 (70.48)	130 (67.01)	0.4317	
	Yes	198 (30.56)	134 (29.52)	64 (32.99)		
Cancer (%)	No	589 (90.90)	413 (90.97)	176 (90.72)	1	
	Yes	59 (9.10)	41 (9.03)	18 (9.28)		
Charlson comorbidity index (median [IQR])		7.000 [5.000, 8.000]	7.000 [5.000, 8.000]	7.000 [5.000, 8.000]	0.7325	Non-norm
SOFA (median [IQR])		4.000 [3.000, 6.000]	4.000 [3.000, 6.000]	4.000 [3.000, 6.000]	0.0125	Non-norm
ApsIII (median [IQR])		49.500 [39.000, 63.000]	50.000 [40.000, 63.750]	48.500 [38.000, 61.750]	0.103	Non-norm
Tracheostomy (%)	No	621 (95.83)	436 (96.04)	185 (95.36)	0.8581	
	Yes	27 (4.17)	18 (3.96)	9 (4.64)		
Invasivevent (%)	No	386 (59.57)	266 (58.59)	120 (61.86)	0.4912	
	Yes	262 (40.43)	188 (41.41)	74 (38.14)		
Positive culture (%)	No	560 (86.42)	393 (86.56)	167 (86.08)	0.9692	
	Yes	88 (13.58)	61 (13.44)	27 (13.92)		

Categorical variables were expressed as numbers with percentages. The continuous variables were expressed as median with quartile (Q1, Q3) or mean ± SD depending on their normality of distribution. IQR, Interquartile range; LOS, length of stay; ICU, intensive care unit; Hospital_expire_flag, the status of the patient upon leaving the hospital; 3d, 3 days; 7d, 7 days; 30d, 30 days; 90d, 90 days; BUN, blood urea nitrogen; WBC, white blood cell; RBC, red blood cell; RDW, red blood cell distribution width; INR, international normalized ratio; PT, prothrombin time; PTT, partial thromboplastin time; SBP, systolic blood pressure; DBP, diastolic blood pressure; MBP, mean blood pressure; SPO2, oxyhemoglobin saturation; SOFA, sequential organ failure assessment; ApsIII, acute physiology score III.

The observed mortality rates were 8.02% (52 cases) at 3 days, 18.67% (121 cases) at 7 days, 33.49% (217 cases) at 30 days, and 38.89% (252 cases) at 90 days. Of the patients, 386 (59.57%) were female, and 262 (40.43%) were male. The median age of the patients was 76.808 years (IQR 64.969, 86.338). Table 4 indicates that the baseline characteristics in the training and testing cohorts were similar in terms of demographics, vital signs, laboratory tests, comorbidities, scoring systems, and other relevant characteristics.

### Selected variables and scoring models

To achieve a good balance between predictive performance and simplicity, we selected the top six predictors from a total of 47 candidate variables for each of the four models (Figures 2, 3). In the 3-day death scoring model, APS-III, white blood cell (WBC), heart rate, platelet count, age and partial thromboplastin time (PTT) were selected. In the 7-day death scoring model, APS-III, age, red cell distribution width (RDW), glucose, PTT, and blood urea nitrogen (BUN) were selected. In the 30-day death scoring model, APS-III, age, Charlson Comorbidity Index (CCI), BUN, RDW, and WBC were selected. In the 90-day death scoring model, ApsIII, age, RDW, BUN, CCI, and glucose were selected. The scoring models calculated from mortality rates are presented in Table 1.

### Performance evaluation

**Figure 5:**
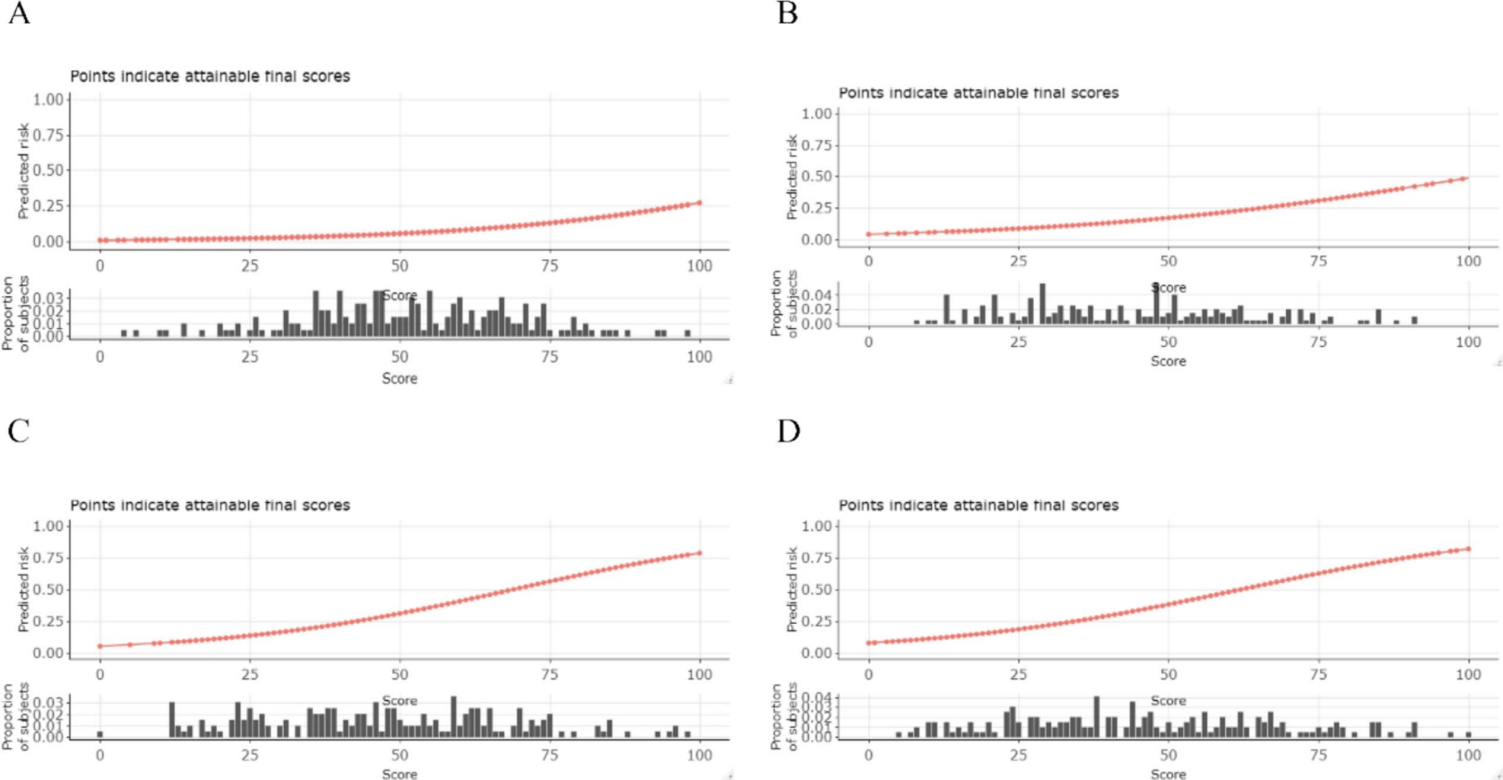
Conversion table and visualization of predicted risk. The sum total score (x-axis) for each outcome can be converted to a probability (y-axis) of achieving the said outcome and histograms display the proportion of our study population that would receive each score. **(A)** 3-day death. **(B)** 7-day death. **(C)** 30-day death. **(D)** 90-day death.

The discriminative performance of the four prognostic models is shown in Figure 4. The AUCs for the 3-day death, 7-day death, 30-day death, and 90-day death in the training cohort were 0.841, 0.800, 0.828, and 0.810, respectively. In the test cohort, the AUCs for 3-day death, 7-day death, 30-day death, and 90-day death were 0.698, 0.678, 0.724, and 0.730, respectively. The predicted risk, patient ratios, and performance indicators (best score threshold, sensitivity, specificity, PPV, NPV) for each scoring model at different scoring intervals based on the testing cohort are shown in Table 2. As predicted risk thresholds increase, accuracy and specificity improve, while sensitivity decreases. Additionally, Table 3 presents the performance indicators based on the best score threshold. Figure 5 illustrates the conversion of scores into the probability of achieving the desired outcome and shows the percentage of the final study population that attained specific scores in the model. The predicted risk increases with higher scores, while the majority of patients’ scores are concentrated in the middle range (between 25 and 75), approaching a normal distribution.

The total score on the x-axis can be converted into the probability of achieving a specific outcome on the y-axis, and histograms illustrate the proportion of our study population that would receive each score.

## Discussion

We analyzed 648 ischemic stroke patients exhibiting moderate to severe consciousness disorders, all of whom were admitted to the ICU, as recorded in the MIMIC-IV database. In recent years, the focus on mortality prediction among ICU patients has grown significantly (19, 20), carrying important implications for improving patient outcomes, optimizing ICU resource utilization, and enhancing the financial efficiency of ICU management systems (21). A study of the stroke population in China indicates that early consciousness disorders should be a critical consideration in the acute management of stroke patients due to their association with increased complications and poorer outcomes (4). The prognostic factors affecting this population warrant further exploration. Therefore, we constructed four risk-scoring models using machine learning techniques specifically for ischemic stroke patients with moderate to severe consciousness disorders. These models demonstrated strong discriminative performance, accurately predicting mortality risk at 3, 7, 30, and 90-day intervals. While our models performed well across all time points, a slight discrepancy was observed between short-term and long-term predictions. The slightly lower AUC values for predicting 3- and 7-day mortality compared to 30-day and 90-day mortality may result from the acute-phase variability and the model’s reliance on baseline admission data. Short-term mortality is often influenced by sudden complications, whereas long-term outcomes are more dependent on stable predictors such as comorbidities and age. In contrast to traditional prognostic models, particularly logistic regression and its derivative nomograms, which are widely used for binary outcomes like mortality, the AutoScore framework provides a more streamlined and automated approach. Traditional logistic regression models require manual variable selection and extensive calibration, often leading to complex nomograms that can be difficult for clinicians to interpret. In comparison, AutoScore leverages methods such as random forest-based ranking and cross-validation to automatically identify a parsimonious set of predictive variables. This not only simplifies the model but also enhances interpretability. More importantly, its straightforward structure enables rapid bedside application, making it especially valuable in clinical settings where ease of use and interpretability are essential. These advantages make AutoScore a compelling choice for clinicians managing high-risk stroke patients in the ICU, as it balances predictive accuracy with practical usability, ultimately improving decision-making and patient care.

Handling numerous potential features derived from electronic health records poses challenges such as model overfitting, poor generalizability to new data, and increased complexity, which can make the model difficult to interpret (15). Therefore, variable selection is essential in model development, as it simplifies predictive models while maintaining accuracy (22). To mitigate the risk of overfitting, several robust strategies were employed within the AutoScore framework (23). First, our dataset was randomly split into training and test sets, and 10-fold cross-validation was implemented during the parsimony plot generation to ensure a stable and reliable variable ranking. This process allowed us to select a minimal yet highly predictive set of variables, reducing model complexity while maintaining predictive power. Additionally, during the weighting step, the framework effectively penalized less predictive variables, further enhancing model simplicity and reliability.

According to our data-driven variable selection process, the APS-III score and age were identified as key variables. The APS-III score assesses disease severity in ICU patients based on physiological variables collected during the first 24 h after admission (24). Recent studies have indicated that the APS-III score plays an important role in predicting the prognosis of ischemic stroke patients (25, 26). Consistent with these findings, our study demonstrated that higher APS-III scores were associated with an increased risk of mortality at 3, 7, 30, and 90 days. Similarly, age is a well-established risk factor for ischemic stroke, with older patients experiencing higher mortality and poorer quality of life compared to younger patients (27). A prospective single-center study on acute ischemic stroke (AIS) patients also found that advancing age is associated with a decline in favorable outcomes and survival after endovascular therapy (28). Additionally, an aging population increases the prevalence of atrial fibrillation, obesity, type 2 diabetes, hypertension, hypercholesterolemia, and coronary artery disease, and acts as an early predictor of fatal infectious complications after a stroke (29). Our models also incorporated several indicators from complete blood counts, blood biochemistry, and coagulation function tests, which are readily accessible in clinical practice. Studies have shown that RDW, WBC, platelet count, and PTT are associated with patient prognosis. A retrospective study demonstrated that higher RDW levels were associated with an increased risk of death, with all-cause mortality rising by 23% for each 1% increment in RDW (30). RDW has also been linked to an elevated risk of mortality from ischemic stroke, cardiovascular disease, cancer, chronic lower respiratory tract disease, and cardiac disorders (30–33). In AIS patients, a higher WBC count on admission is an independent predictor of stroke severity at admission, a greater degree of disability at discharge, and 30-day mortality (34). Likewise, a 10-year follow-up study found that patients with elevated WBC levels at stroke onset had a significantly higher risk of subsequent vascular events and mortality, even after adjusting for other risk factors (35). Another study showed that increasing WBC count predicted poor outcomes and mortality in stroke patients treated with intravenous thrombolysis (IVT) (36). Regarding platelet count, a systematic review found that thrombocytopenia, present in 8.3–67.6% of ICU admissions, is associated with high illness severity, sepsis, organ dysfunction, and an increased risk of death (37). Furlan et al. found that both thrombocytopenia and thrombocytosis upon initial admission are associated with higher mortality after AIS (38). Studies have shown that higher INR and PTT in AIS patients are associated with worse NIHSS scores, indicating that changes in coagulation parameters may negatively impact stroke prognosis (39, 40). Additionally, a study involving 3,355 AIS patients found that higher BUN levels at admission were significantly linked to increased all-cause in-hospital mortality (41). Moreover, elevated blood glucose levels at admission have been shown to predict higher short-term mortality in acute cerebral ischemia, emphasizing the importance of early glucose control (42). It has also been confirmed that elevated admission glucose levels are associated with higher mortality and morbidity in stroke patients, reinforcing the need for effective glucose management in this population (43). Furthermore, a higher heart rate at admission has been linked to an increased risk of stroke recurrence and mortality, highlighting the critical role of heart rate management in patients with AIS and atrial fibrillation (44). A study by Goldstein et al. reported that the CCI at admission is significantly associated with the prognosis of ischemic stroke patients (45). The study emphasizes the necessity of comorbidity adjustment in stroke outcome research, revealing that higher CCI scores at admission are correlated with increased one-year mortality rates.

The selection of variables and model construction are consistent with established clinical principles, and these findings merit further investigation by healthcare professionals. In the short-term mortality prediction models, the inclusion of variables such as WBC, heart rate, PTT, and platelet count reflects the critical condition of these patients at admission. Future research should incorporate more critically ill stroke patients to continuously optimize the algorithm and establish appropriate thresholds for stratifying the population into various risk categories. In the relatively long-term mortality prediction models, the inclusion of variables such as the CCI, BUN, and blood glucose levels highlights the complexity of these patients’ conditions at admission. Whether scientific, standardized, and precise chronic disease management before and after ischemic stroke onset benefits the relatively long-term prognosis of these patients requires further validation through prospective studies. It is noteworthy that, compared to in-hospital and short-term mortality, relatively long-term mortality is significantly elevated. This indicates that a considerable number of patient deaths occur post-discharge, highlighting a cohort whose health issues warrant significant attention from healthcare providers in the future (46). In the high-pressure and fast-paced clinical environment, emergency and neurology healthcare professionals often rely on subjective personal experience and clinical judgment when assessing critically ill patients. However, our data-driven predictive model provides an objective, convenient, and practical visual scoring tool. It aids in the rapid identification of patients with moderate to severe consciousness disorders due to ischemic stroke who are at high risk of early mortality. This allows healthcare providers to receive early warnings and develop targeted medical plans and care strategies, while also preparing families psychologically. Moreover, it facilitates personalized assessments of relatively long-term mortality risks for these patients, providing references for both medical professionals and patients in prognostic evaluations, thereby promoting more efficient utilization of medical resources.

However, several limitations should be acknowledged. First, given the limited follow-up information available in the MIMIC database, this study focused exclusively on mortality rates and did not examine the sequelae or long-term outcomes of ischemic stroke in these patients. After discharge, many of these patients experience varying degrees of functional impairment and may also develop psychological issues, leading to a reduced quality of life. Second, we did not include NIHSS scores (47) in our study due to the high proportion of missing values in the MIMIC database. Including only patients with available NIHSS scores would have led to significant data loss and potential selection bias (48). In the future, natural language processing (NLP) techniques could be utilized to indirectly obtain missing NIHSS scores from clinical text records. Preliminary analyses using NLP techniques have shown promise, though challenges remain in ensuring accuracy and consistency (49). Future work will focus on validating the accuracy of NLP-derived NIHSS scores by comparing them with manually annotated records. Additionally, ischemic stroke patients with consciousness disorders typically have higher NIHSS scores. Training a model on such data may limit its generalizability, particularly when applied to patients with lower NIHSS scores, potentially introducing sample bias. Third, this study included only hospitalized patients, potentially excluding a significant proportion of individuals who either died before reaching the hospital or did not seek hospitalization. This limitation may affect the generalizability of the model. To address this limitation, future research could incorporate data from emergency services or autopsy records to provide a more comprehensive view of stroke outcomes. Finally, although we performed internal validation through random splits of the dataset, the lack of external validation remains a limitation of this study. In future work, we plan to address this by using future releases of the MIMIC database for temporal validation and incorporating prospective multicenter study data for external validation.

## Conclusion

In this study, we identified prognostic factors for ischemic stroke patients with moderate to severe consciousness disorders and developed a data-driven clinical scoring tool using machine learning algorithms. This tool can assist healthcare professionals in objectively assessing both short-term and relatively long-term mortality risks in these patients.

## Data Availability

The raw data supporting the conclusions of this article will be made available by the authors, without undue reservation.
